# Resolving the Brainstem Contributions to Attentional Analgesia

**DOI:** 10.1523/JNEUROSCI.2193-16.2016

**Published:** 2017-03-01

**Authors:** Jonathan C.W. Brooks, Wendy-Elizabeth Davies, Anthony E. Pickering

**Affiliations:** ^1^Clinical Research Imaging Centre and; ^2^School of Physiology, Pharmacology and Neuroscience, University of Bristol, Bristol, BS8 1TD, United Kingdom, and; ^3^Department of Anaesthesia, University Hospitals Bristol, Bristol, BS2 8HW, United Kingdom

**Keywords:** attention, brainstem, endogenous analgesia, locus coeruleus, periaqueductal gray, rostral ventromedial medulla

## Abstract

Previous human imaging studies manipulating attention or expectancy have identified the periaqueductal gray (PAG) as a key brainstem structure implicated in endogenous analgesia. However, animal studies indicate that PAG analgesia is mediated largely via caudal brainstem structures, such as the rostral ventromedial medulla (RVM) and locus coeruleus (LC). To identify their involvement in endogenous analgesia, we used brainstem optimized, whole-brain imaging to record responses to concurrent thermal stimulation (left forearm) and visual attention tasks of titrated difficulty in 20 healthy subjects. The PAG, LC, and RVM were anatomically discriminated using a probabilistic atlas. Pain ratings disclosed the anticipated analgesic interaction between task difficulty and pain intensity (*p* < 0.001). Main effects of noxious thermal stimulation were observed across several brain regions, including operculoinsular, primary somatosensory, and cingulate cortices, whereas hard task difficulty was represented in anterior insular, parietal, and prefrontal cortices. Permutation testing within the brainstem nuclei revealed the following: main effects of task in dorsal PAG and right LC; and main effect of temperature in RVM and a task × temperature interaction in right LC. Intrasubject regression revealed a distributed network of supratentorial brain regions and the RVM whose activity was linearly related to pain intensity. Intersubject analgesia scores correlated to activity within a distinct region of the RVM alone. These results identify distinct roles for a brainstem triumvirate in attentional analgesia: with the PAG activated by attentional load; specific RVM regions showing pronociceptive and antinociceptive processes (in line with previous animal studies); and the LC showing lateralized activity during conflicting attentional demands.

**SIGNIFICANCE STATEMENT** Attention modulates pain intensity, and human studies have identified roles for a network of forebrain structures plus the periaqueductal gray (PAG). Animal data indicate that the PAG acts via caudal brainstem structures to control nociception. We investigated this issue within an attentional analgesia paradigm with brainstem-optimized fMRI and analysis using a probabilistic brainstem atlas. We find pain intensity encoding in several forebrain structures, including the insula and attentional activation of the PAG. Discrete regions of the rostral ventromedial medulla bidirectionally influence pain perception, and locus coeruleus activity mirrors the interaction between attention and nociception. This approach has enabled the resolution of contributions from a hub of key brainstem structures to endogenous analgesia.

## Introduction

Pain is a subjective, multidimensional, emotional experience whose characteristic is strongly dependent upon behavioral context (Melzack et al., 1982). Cognitive processes are known to modulate pain perception (Tracey and Mantyh, 2007; Bushnell et al., 2013). Examples include the following: distraction-based analgesia (Miron et al., 1989), stress analgesia (Butler and Finn, 2009), or negative mood increasing pain perception (Villemure and Bushnell, 2002). These cognitive/psychological factors are of particular importance in patients with chronic pain conditions (Bushnell et al., 2013). Previous imaging studies have identified forebrain structures whose activity is related to pain modulation in the following contexts: perceived control (Wiech et al., 2006), placebo responses (Petrovic et al., 2002; Wager et al., 2004; Bingel et al., 2011), hypnotic suggestion (Rainville et al., 1999), and attention/distraction (Bushnell et al., 1985; Peyron et al., 1999; Bantick et al., 2002; Brooks et al., 2002; Valet et al., 2004). Notably, activity with the rostral anterior cingulate cortex (rACC) and mPFC is context sensitive and appears to be causally involved in pain suppression (Petrovic et al., 2002; Wager et al., 2004; Eippert et al., 2009).

It is proposed that these cortical areas suppress nociceptive signaling through projections to midbrain structures, such as the periaqueductal gray (PAG). Increased functional connectivity between the PAG and forebrain has been observed during attentional modulation of pain and placebo responses (Petrovic et al., 2002; Lorenz et al., 2003; Valet et al., 2004; Eippert et al., 2009). PAG activity has also been shown to be increased by distraction from the noxious stimulus, and this correlated with the degree of analgesia (Tracey et al., 2002), providing some evidence for top-down cognitive modulation of pain. Further, attentional modulation of spinal BOLD responses to a nociceptive stimulus indicates that the analgesic effect involves descending control of spinal processing (Sprenger et al., 2012).

There is an extensive body of evidence from both animal studies and human investigations linking the PAG with behavioral integration and endogenous analgesia (Carrive and Morgan, 2012; Linnman et al., 2012). However, this analgesic action is predominantly mediated through activation of brainstem centers, such as the rostral ventromedial medulla (RVM) and the locus coeruleus (LC), which themselves send descending projections to the spinal dorsal horn to modulate nociception (Fields, 2000; Ossipov et al., 2010). Interestingly, animal investigations have shown that both of these structures contain neurons that are activated by painful stimuli (Cedarbaum and Aghajanian, 1978; Heinricher et al., 1989), can exert bidirectional influence on nociception (Zhuo and Gebhart, 1992; Hickey et al., 2014), and, in the case of the LC, have a prominent role in attentional processing (Aston-Jones and Cohen, 2005; Sara, 2009). We therefore anticipated that they may show activity in different domains of a cognitive pain modulation task.

To date, most imaging studies looking at descending pain control have focused on the role of the PAG in pain perception and have not reported on lower brainstem activity. The RVM and LC are rarely reported in the pain functional imaging literature, mostly because of their size and location, but also because accurate localization of signal is hindered through the lack of comprehensive probabilistic brainstem atlases. Furthermore, brainstem imaging suffers from an increased contribution of confounding signals, such as physiological noise (Astafiev et al., 2010; Brooks et al., 2013), and image distortion due to bulk susceptibility (Cooke et al., 2004).

In this study, we have addressed this deficit in our knowledge by asking the following questions: (1) which brainstem structures are involved in descending pain control, and is their activity related to perceived pain? and (2) what cortical structures represent these processes? We used an attention-based analgesia paradigm, which we predicted would recruit circuits involved in descending pain control (Bushnell et al., 1984; Tracey et al., 2002; Villemure and Bushnell, 2002). By recording pain ratings during high-resolution functional imaging, we were able to identify brain regions whose activity tracks pain perception, and determine which brainstem structures are implicated in analgesia.

## Materials and Methods

Subjects were recruited using poster and E-mail advertisements at the University of Bristol, and 28 subjects were screened (two declined to be involved in the study and one had a metal implant so was excluded; four were unable to give consistent responses to thermal stimuli so were excluded; and one dropped out of the study before the examination session). Twenty right-handed (verified with the Edinburgh Handedness inventory) (Oldfield, 1971), healthy subjects (median age, 25 years, range 18–51 years; 10 females) participated in the whole study, which had approval from the University of Bristol Faculty of Science Human Research Ethics Committee (reference 280612567).

Normal inclusion/exclusion criteria for participation in MRI studies were applied during screening. The presence of significant medical or psychiatric disorder (including depression) or pregnancy precluded participation. Subjects with a chronic pain condition or those who were regularly taking analgesic or psychoactive medications were excluded from the study.

Subjects attended for two sessions: during an initial consent visit, they were screened for participation and task difficulty and thermal stimulation levels were defined by titration for each subject (calibration); subsequently, they returned for their fMRI scan with the calibrated stimuli (examination).

During the calibration session, a condensed version of the experimental paradigm was run for each participant outside the scanner environment to familiarize subjects with the protocol and define suitable stimulation parameters. Thermal stimuli were delivered to the left volar forearm (C6 dermatome) using a circular contact thermode with surface area 573 mm^2^ (CHEPS Pathway, MEDOC) with the baseline temperature matching skin temperature (32°C). For each subject, the thermode temperature was adjusted in a pseudo-random sequence to identify a stimulus level that produced a pain rating of 6 of 10 using the method of limits (Moloney et al., 2012). The pain rating was provided verbally using a numerical rating scale, with 0 corresponding to “no pain” and 10 to the “worst pain imaginable.” Thermal stimuli lasted 30 s, with the TARGET temperature set to either 36°C (innocuous heat, “low”) or 42°C–45°C (noxious, “high”), onto which were superimposed brief (1 s) temperature spikes of 2°C, 3°C, and 4°C above TARGET (after Valet et al., 2004). This heating profile was used to maintain painful perception while avoiding skin sensitization.

**Figure 1.**
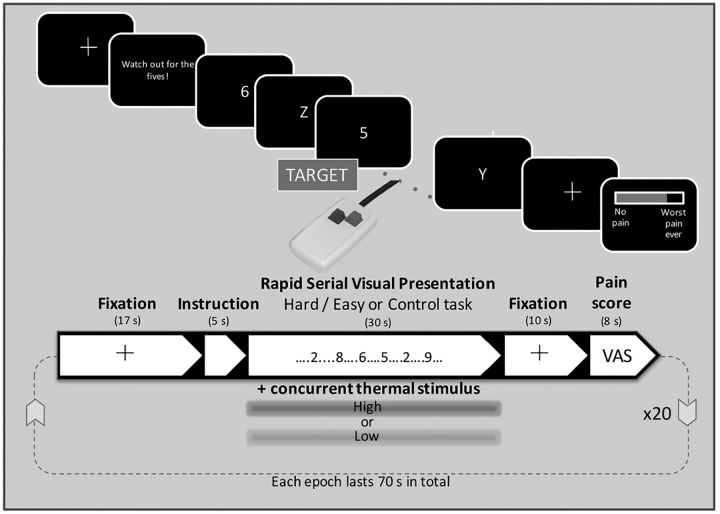
For the RSVP task (shown on the top half of the figure), the subject attends for the presence of the target (“5”) and responds via the button box while inhibiting responses to all other distractor characters. The speed of presentation of the characters was varied to titrate the task difficulty for each subject. Following a rest period and a cue, the RSVP was presented concurrently with thermal stimulation of the left volar forearm, delivered via a CHEPS thermode. Ten seconds after the end of each task/thermal stimulation period, the subject used the button box to provide a pain intensity rating. The experiment used a 2 × 2 factorial design (high|low temperature, hard|easy task), and a control condition (high temperature, no distractors), with 4 repetitions of each condition, giving 20 blocks in total.

To avoid some limitations of earlier studies using, for example, STROOP or *n*-back tasks, where perceived difficulty (and therefore arousal) can vary dramatically between individuals, we used the rapid serial visual presentation (RSVP) task to manipulate attention (Potter and Levy, 1969). The task (Fig. 1) was programmed in Presentation software (Neuro-Behavioral Systems). During the RSVP task, a stream of letters and numbers are presented sequentially in the center of the screen. The subject is instructed to respond rapidly with a button press whenever they spot the target character (“5”) while inhibiting responses to the distractor characters. During the calibration phase, the intercharacter interval of RSVP task was systematically altered from the shortest 32 ms to longest 192 ms gap. Subjects' task performance was recorded to determine the “speed” at which their detection rate was 70%, adjusted for hits, misses, and false alarms. This speed was subsequently used for the “hard” RSVP task. The speed of the “easy” RSVP task was set at either 192 or 256 ms (if the “hard” task interval for the subject was <100 ms or >100 ms, respectively), corresponding to a performance level of >90% correct. A third minimal attention load (“control”) condition was also used with “5” presented at the lowest speed (256 ms) with a cross (“+”) interspersed (i.e., in the absence of any distracters). Task performance during the MRI scanning session was assessed by calculating *d*′ (Green and Swets, 1966).

### 

#### 

##### Data acquisition.

Imaging was performed with a 3T Skyra MR system (Siemens Medical Solutions) and 32-channel receive-only head coil. Subjects were instructed to remain as still as possible during scanning, and head motion minimized by placing memory foam padding beside their head. Following acquisition of 3-plane localizer images, T1- and T2-weighted structural scans were acquired for the purpose of spatial normalization and brainstem atlas development, respectively. A sagittal T1-weighted volume scan was acquired with MPRAGE pulse sequence with the following parameters: TE/TI/TR = 2.25/800/1900 ms, flip angle = 9**°**, averages = 2, GRAPPA acceleration factor = 2, resolution 0.94 × 0.94 × 0.9 mm, and a sagittal T2-weighted volume scan with SPACE (Sampling Perfection with Application optimized Contrasts using different flip angle Evolution) pulse sequence with the following parameters: TE/TI/TR = 388/1800/5000 ms, variable flip angle, GRAPPA acceleration factor = 2, resolution 0.45 × 0.45 × 0.9 mm. The T2-weighted sagittal volume scan was prescribed with its *y*-axis (i.e., superior-inferior) parallel to the floor of the fourth ventricle.

Functional data were acquired with a BOLD-sensitive EPI sequence (gradient echo EPI, TE/TR = 30/3000 ms, flip angle = 80**°**, GRAPPA acceleration factor = 2, resolution 1.5 × 1.5 × 3.5 mm). The axial-oblique slices were prescribed to be perpendicular to the floor of the fourth ventricle to optimally capture signal from the LC and other brainstem nuclei, which tend to lie parallel to the long-axis of the brainstem. Last, a gradient echo B_0_ field mapping sequence was acquired with the following sequence parameters: TE1/TE2/TR = 4.92/7.38/520 ms, flip angle 60**°**, resolution = 3 × 3 × 3 mm.

During scanning, subjects' cardiac pulse waveform and respiratory movements were monitored via a pulse oximeter and respiratory bellows, respectively. Physiological data were acquired with standard clinical monitoring equipment (Expression MRI Monitoring System, InVivo), and the analog signals and scanner volume triggers were recorded on an MP150 data acquisition system (BIOPAC) at a sampling rate of 100 Hz.

##### Examination protocol.

The protocol followed a 2 × 2 (+1) factorial block design with thermal stimulus type (high vs low) and RSVP difficulty (hard vs easy) varied systematically between blocks with an additional control task with high thermal stimulation only. Each stimulus combination was repeated 4 times giving a total of 20 blocks. Immediately before each stimulation block subjects were given a 5-s-long cue to prepare for stimulation, but no indication was given as to the type of stimulus they would receive. In between stimuli, a cross was presented at the center of the screen. A summary of the experimental design can be seen in Figure 1. The functional imaging experiment lasted 26 min, and the whole scanning session typically lasted less than 1 h.

During functional imaging, visual stimuli were presented using rear-projection on to a screen in the scanner bore and visible to subjects via a mirror mounted on the head coil. Stimulus presentation and timing were synchronized to the scanner trigger to minimize timing errors. Thermal stimuli were delivered simultaneously with the RSVP task, and each lasted 30 s. Subjects indicated the presence of the visual target stimulus (“5”) by pressing on a button box held in their right hand with their index finger (Lumina LP-400, Cedrus), and their responses were recorded on Presentation software. Subjects were instructed to respond to the RSVP task as quickly as they could, without sacrificing accuracy. Following each thermal stimulus, after a gap of 10 s, an interactive visual analog scale (VAS) was displayed on the projection screen for 8 s, and subjects provided average pain intensity ratings for that stimulus using the button box. By pressing the buttons with middle or index fingers, subjects moved a sliding-marker on the VAS (from “no pain” to “worst pain imaginable”). Tick marks and numbers (0–10) were positioned below the scale to assist rating.

##### Data analysis.

Behavioral data recorded during scanning (pain VAS, RSVP task performance) and the effect of experimental condition were examined using ANOVA in SPSS software (SPSS version 23, IBM). Following conversion from DICOM to Nifti (dcm2nii, https://www.nitrc.org/plugins/mwiki/index.php/dcm2nii:MainPage), functional imaging data were analyzed with FMRIB's Software Library version 5.0.8.1 (Jenkinson et al., 2012). Individual subjects' data were motion corrected by realigning each volume to the midpoint time series volume using MCFLIRT (Jenkinson et al., 2002). Each was then coregistered to the high-resolution T1-weighted structural scan using a combination of fieldmap based unwarping using FUGUE (Jenkinson, 2003), boundary-based registration (Greve and Fischl, 2009), and FLIRT (Jenkinson and Smith, 2001). Subsequently, functional data were spatially smoothed with a kernel size of 3 mm (FWHM), and high pass temporally filtered (cutoff 120 s). Whereas others have found it necessary to use manual registration and affine transformation to achieve good alignment between EPI data of the brainstem and the subject's T1-weighted structural scan (Napadow et al., 2006), we found that the fieldmap unwarping made such manual intervention unnecessary.

To assess attention-mediated analgesia, activity in each of the 5 conditions (easy|high, hard|high, easy|low, hard|low, control|high) and tasks of no interest (cue, rating period) were estimated using a hemodynamic response function (gamma basis function, σ = 3 s, mean lag = 6 s) by using a GLM incorporating local autocorrelation correction (FILM), (Woolrich et al., 2001). The full design included temporal derivatives and a slice-wise physiological noise model (Brooks et al., 2008; Harvey et al., 2008).

In a second analysis, the relationship between perceived pain intensity and BOLD signal was estimated via an intrasubject parametric regression model incorporating subjective pain ratings for each of the 20 stimuli experienced. A constant regressor (weighting = 1 for all 20 stimuli) to model the average activity across conditions, nuisance regressors (cue, rating period), and the physiological noise model were also included and estimated with FEAT. All regressors were inherently orthogonalized with respect to each other.

Parameter estimate maps were transformed into the space of the MNI “standard” brain (MNI152), using a combination of affine transformations (FLIRT) and nonlinear warping (FNIRT) with warp spacing of 5 mm. Of particular concern when studying the brainstem is the ability to bring functional imaging data into alignment with the chosen template. Previously, researchers have used unbiased templates to achieve excellent results in the cerebellum (Diedrichsen, 2006). By combining field map unwarping and nonlinear registration to a template, itself derived using nonlinear transformations (the MNI 152 nonlinear sixth generation atlas), we could bring all subjects into good alignment in the brainstem. This was assessed by visual inspection of the average functional image (“mean_func.nii.gz”) obtained after registration to MNI space, which retained sharp intensity boundaries, and matched closely the anatomy of the brainstem on the MNI template.

**Figure 2.**
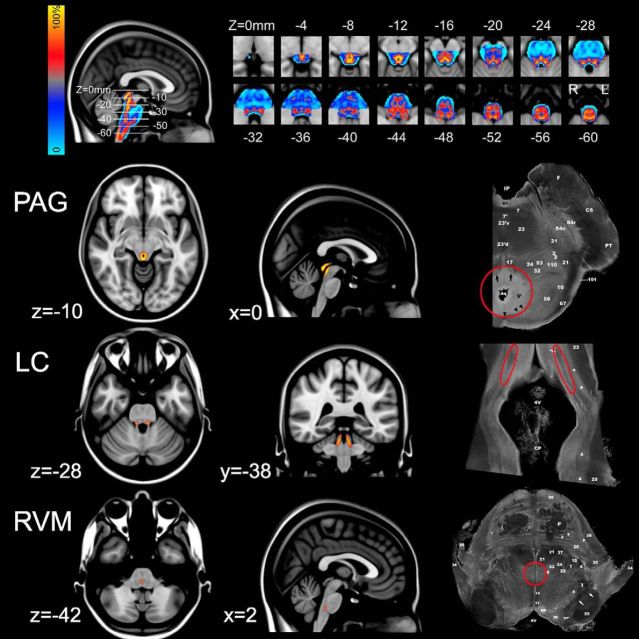
Creation of probabilistic brainstem atlas. T2-weighted volumetric images acquired from the 20 healthy subjects were normalized (using the DARTEL technique) and segmented (using the VBM8 toolbox) into gray matter, white matter, or CSF. The gray matter probability maps were registered to one another, to create a probabilistic gray matter atlas (see top row). The color bar represents the probability of a given voxel being gray matter. The main areas of interest were the PAG, LC, and RVM, which were identified by thresholding the atlas at *p* > 0.7 (i.e., >70% chance of being gray matter) and then outlining the structures of interest on the basis of comparison to known anatomical landmarks taken from the Duvernoy brainstem atlas. Sections shown on the right hand side with structures of interest indicated by red circles (Naidich et al., 2009). All slice locations are given in the MNI coordinates.

To aid identification of brainstem nuclei, a gray matter probability map was constructed using the DARTEL (Diffeomorphic Anatomical Registration Through Exponentiated Lie algebra) spatial normalization technique available in SPM8 (Ashburner, 2007) running in MATLAB R2015a software (The MathWorks). Briefly, T2-weighted volumetric data were segmented using the VBM8 toolbox (http://dbm.neuro.uni-jena.de/vbm8) into gray, white, CSF, and other tissue types, and the segmented gray matter maps registered to one another using the DARTEL algorithm. The final result is a probabilistic template specific to the study group, which was then transformed into the space of the MNI atlas. With the threshold for the probabilistic map set at *p* = 0.7 (i.e., at least 70% gray matter), masks were defined for the PAG, RVM, and LC taking advantage of the inherent high contrast between the gray and white matter structures of the brainstem (see Fig. 2). These were validated with reference to anatomical sections on a human brainstem atlas (Naidich et al., 2009).

Group responses were estimated in two ways. The first used a whole-brain analysis, with a 2 × 2 repeated-measures ANOVA mixed-effects model in FEAT using FLAME (Stages 1 and 2), using cluster based correction for inference (height threshold *Z* > 3.09, corrected cluster extent threshold *p* < 0.05). Main effects (task difficulty or temperature) and their interaction (task × temperature) were explored through signed contrasts, where positive implied high > low temperature and hard > easy task difficulty (and vice versa) (e.g., positive main effect of temperature, [hard|high + easy|high] > [hard|low + easy|low]). Similarly, a whole-brain mixed-effects analysis with a one-sample *t* test was used to explore brain regions in which the slope of the pain rating versus BOLD relationship (intrasubject parametric model) was non-zero across the group (height threshold *Z* > 3.09, corrected cluster extent threshold *p* < 0.05). The second approach used nonparametric permutation testing (RANDOMISE) (Nichols and Holmes, 2002) with anatomical masks for specific brainstem nuclei hypothesized (a priori) to be activated during attention-mediated analgesia. Activity within brainstem nuclei was assessed using these probabilistic masks and permutation testing, to test for main effects or an interaction, and are reported using a threshold free cluster enhancement (TFCE) corrected *p* < 0.05. Data from the parametric regression model were also subjected to the same masked analysis.

Last, the magnitude of attention-mediated analgesia (ΔVAS), defined as the difference in average pain ratings between the two task difficulties (easy − hard) during high temperature stimulation, was computed. We tested whether differences in parameter estimates (ΔBOLD) predicted individual analgesia (ΔVAS) across the group in an intersubject regression model. Whole-brain analysis was performed as described above using a paired *t* test (hard|high, easy|high), which examined whether the magnitude of differences between conditions could be explained by the difference in associated pain ratings. For the brainstem, between-subject differences (i.e., hard|high − easy|high) in BOLD were modeled with the demeaned subject specific ΔVAS ratings. The spatial location of voxels whose activity predicted the magnitude of analgesic effect was determined using permutation testing within the previously defined probabilistic brainstem anatomical masks. Results are reported with TFCE corrected *p* < 0.05.

## Results

### Behavioral data

**Figure 3.**
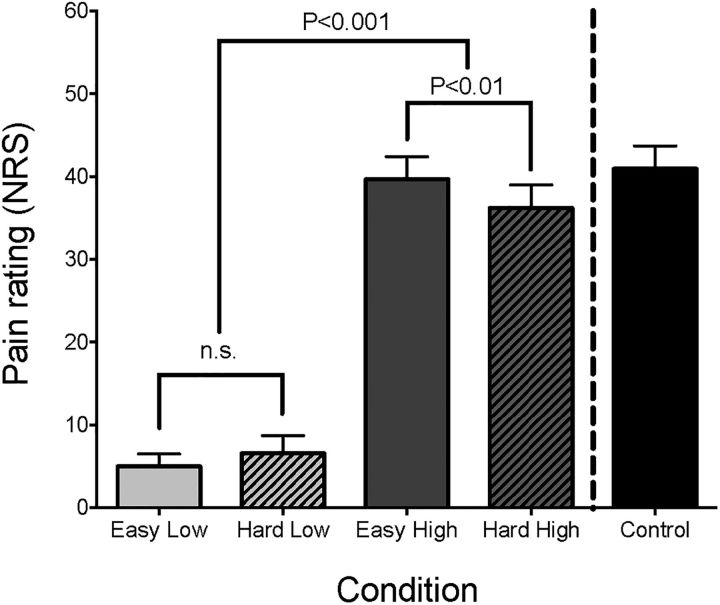
Behavioral data acquired during scanning. The average pain intensity ratings for each of the five experimental conditions (easy|low, hard|low, easy|high, hard|high, no-distractor|high) are shown, along with error bars representing the SEM. A 2 × 2 repeated-measures ANOVA (excluding the control condition) demonstrated a significant main effect of temperature, and a temperature × task interaction. *Post hoc* paired *t* tests indicated that this was due to a significant reduction in pain ratings when subjects experienced high temperature stimulation while performing the RSVP task at their “hard” speed, compared with identical temperature stimulation with an easy (i.e., slow) RSVP task.

The average high temperature was 44.2°C (range 42°C-45°C), and the temperature in the low condition was always 36°C. The range of intervals used for character presentation during the hard task condition was 48 to 160 ms (mode = 80 ms), whereas for the easy condition only 6 subjects used the slower interval of 256 ms. The corresponding pain ratings for the four conditions of the factorial design and the control block are shown in Figure 3. This revealed the expected main effect of temperature (*p* < 0.001) and a task × temperature interaction (*p* < 0.01, repeated-measures ANOVA). *Post hoc* analysis showed that the interaction was due to a difference in pain ratings during the high temperature condition (mean high|easy = 39.7, SEM 2.7; mean high|hard = 36.2, SEM 2.8; *p* < 0.01, paired *t* test), indicating an analgesic effect of performing the hard task. As expected, there was a trend for a difference in RSVP task performance *d*′ (main effect of task, *p* = 0.056), but no effect of temperature (main effect of temperature *p* = 0.146) and no interaction (*p* = 0.832).

### Attention-mediated analgesia (whole-brain corrected)

**Figure 4.**
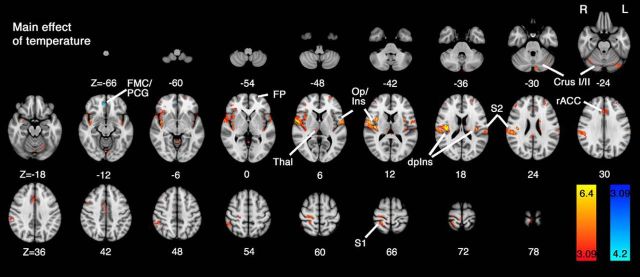
Whole-brain mixed-effects analysis of the main effect of temperature. Activity in response to high > low temperature stimuli revealed a widespread network of cortical and subcortical regions, as has been previously demonstrated in response to painful thermal stimulation (Apkarian et al., 2005). In particular, contralateral activity was observed in the dorsal posterior insula (dpIns), S1, S2, and thalamus (Thal). Further regions activated include the rostral anterior cingulate cortex (rACC), frontal pole (FP), and Crus I in the cerebellum. A negative main effect of temperature (low > high) was observed in a region overlapping frontal medial cortex (FMC)/paracingulate gyrus (PCG). Data were obtained from cluster-based thresholding using an initial threshold of *Z* > 3.09 and corrected significance level of *p* < 0.05.

**Figure 5.**
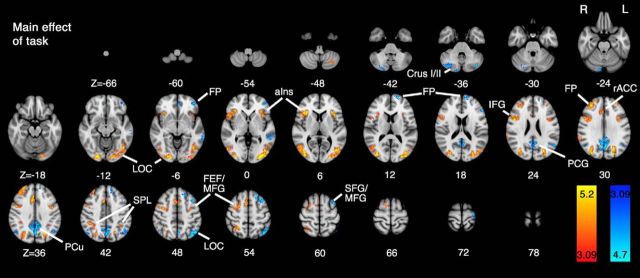
Whole-brain mixed-effects analysis of the main effect of task. The positive response to task (hard > easy) produced activity areas known to be involved in visual information processing and attention (Petersen and Posner, 2012). The task-activated visual association (lateral occipital cortex [LOC]) cortices in the occipital lobes, as well as parietal and frontal regions involved in attention: superior parietal lobule (SPL), anterior insular cortex (aIns), frontal pole (FP), middle frontal gyrus (MFG), inferior frontal gyrus (IFG), PCG, and frontal eye fields (FEF). Task-negative activity was observed in precuneus (PCu), FP, LOC, superior frontal gyrus (SFG), and cerebellum bilaterally. Data were obtained from cluster-based thresholding using an initial threshold of *Z* > 3.09 and corrected significance level of *p* < 0.05.

Group results for the mixed-effects repeated-measures ANOVA imaging experiment are shown in Figures 4 and 5, for the main effects of temperature and task, respectively. Increased activity was observed in the high (painful) versus low (innocuous) contrast across the expected range of cortical and subcortical regions (Apkarian et al., 2005). The most prominent area of activation was centered on the dorsal posterior insula (dpIns) contralateral to the side of stimulation, but was also present in operculoinsular regions bilaterally. Other foci of contralateral activity were observed within the thalamus, primary and second somatosensory areas (S1, S2). Activity was also observed in the rACC, frontal pole, bilateral Crus I, and anterior lobes of the cerebellum. The only area showing increased activity during the reverse contrast, low > high, was a small region in the ventromedial prefrontal cortex: frontal medial cortex (FMC)/paracingulate gyrus (PCG).

For the main effects of task (Fig. 5), the contrast: hard > easy revealed increased activity over an extended area of cerebrum and cerebellum bilaterally, including the following: primary visual and association cortices, anterior insular cortices, frontal eye fields, precentral gyri, frontal pole, and superior and inferior frontal gyri. For the reverse task contrast: easy > hard, activity was mostly confined to a large area of the precuneus, lateral occipital cortex, middle frontal gyri, and frontal pole.

No activation was observed for the interaction contrast at the specified threshold (cluster forming threshold *Z* > 3.09, FWE corrected *p* < 0.05).

Coordinates, *Z* scores, and anatomical location for activated clusters for the different contrasts of main effects are summarized in Table 1.

**Table 1.** Data from analysis of main effects of temperature and task during distraction-based analgesia obtained with cluster-forming threshold *Z* > 3.09 and (corrected) *p* < 0.05*^a^*

Voxels	Maximum *Z*	*x* (mm)	*y* (mm)	*z* (mm)	Atlas label(s)
Positive main effect of temperature (i.e., high > low)					
2281	8.75	40	−16	18	Insular cortex (12.8%); central opercular cortex (16.5%)
362	5.3	22	−44	70	Precentral gyrus (17.7%); postcentral gyrus (23.2%); superior parietal lobule (17.3%)
299	5.94	−54	−4	6	Precentral gyrus (14.9%); central opercular cortex (19.6%)
264	4.34	0	24	30	Paracingulate gyrus (24.2%); cingulate gyrus, anterior division (52.7%)
140	4.16	48	−46	56	Supramarginal gyrus, posterior division (38.0%); angular gyrus (21.4%)
119	4.09	2	−76	−12	Lingual gyrus (18.6%); Cerebellum: left VI (23.4%); vermis VI (16.1%)
114	5.52	−36	−20	16	Insular cortex (27.8%); central opercular cortex (20.5%)
110	4.27	−58	−24	16	Postcentral gyrus (16.5%); supramarginal gyrus, anterior division (15.8%); central opercular cortex (12.6%); parietal operculum cortex (23.3%); planum temporale (10.8%)
89	4.26	2	−32	78	Precentral gyrus (16.2%); postcentral gyrus (23.5%)
87	4.11	−40	−76	−26	Left Crus I (94.5%)
75	4.37	−20	−24	22	Left lateral ventricle (25.8%); left caudate (25.5%)
70	4.58	0	−48	−4	Cerebellum: Left I-IV (25.8%); right I-IV (19.6%)
68	4.04	−28	50	20	Frontal pole (80.6%)
57	3.94	42	42	26	Frontal pole (70.2%)
55	4.04	40	42	0	Frontal pole (63.0%)
51	4.11	−30	64	8	Frontal pole (76.2%)
48	4.47	−40	−6	−10	Insular cortex (55.6%)
47	4.02	10	−20	10	Right thalamus (89.7%)
45	4.61	−10	−82	−30	Left Crus I (23.0%); left Crus II (71.4%)
39	4.26	4	−44	20	Cingulate gyrus, posterior division (63.4%)
37	4.44	−34	8	10	Insular cortex (33.4%); central opercular cortex (22.9%)
35	4.32	4	−26	8	Right thalamus (29.3%)
35	4.03	42	12	44	Middle frontal gyrus (38.5%)
33	4.04	42	−56	−24	Cerebellum: Right VI (42.5%); right Crus I (43.5%)
29	4.32	−24	−70	−26	Cerebellum: Left VI (74.8%); left Crus I (24.2%)
Negative main effect of temperature (i.e., low > high)					
45	4.16	6	44	−12	Frontal medial cortex (40.1%); paracingulate gyrus (35.8%)
Positive main effect of task (i.e., hard > easy)					
1903	5.85	−36	−88	10	Lateral occipital cortex, superior division (17.9%); lateral occipital cortex, inferior division (27.6%)
1772	6.13	24	−88	−12	Lateral occipital cortex, superior division (19.8%); lateral occipital cortex, inferior division (19.4%)
838	5.47	46	6	24	Middle frontal gyrus (11.2%); inferior frontal gyrus, pars opercularis (12.8%); precentral gyrus (23.4%)
567	5.8	4	26	34	Superior frontal gyrus (11.4%); paracingulate gyrus (33.0%); cingulate gyrus, anterior division (19.5%)
513	6.34	32	24	6	Insular cortex (14.7%); frontal orbital cortex (11.2%); frontal operculum cortex (23.6%)
493	6.13	32	48	34	Frontal pole (56.8%); middle frontal gyrus (12.6%)
273	5.71	−34	22	6	Insular cortex (23.8%); frontal orbital cortex (10.8%); frontal operculum cortex (26.8%)
171	4.48	−44	−2	36	Middle frontal gyrus (11.1%); inferior frontal gyrus; pars opercularis (12.2%); precentral gyrus (26.7%)
71	4.15	68	−32	20	Supramarginal gyrus, posterior division (38.3%); angular gyrus (11.2%)
55	4.13	−34	−2	52	Middle frontal gyrus (19.3%); precentral gyrus (31.6%)
43	4.37	−32	48	30	Frontal pole (79.5%)
42	4.25	−38	−46	42	Superior parietal lobule (22.4%); supramarginal gyrus; posterior division (26.6%)
41	4.2	−28	−68	−48	Left Crus II (15.6%); Cerebellum: left VIIb (58.2%)
35	3.93	−52	28	16	Middle frontal gyrus (12.9%); inferior frontal gyrus; pars triangularis (45.4%)
Negative main effect of task (i.e., easy > hard)					
1272	5.62	0	−64	40	Cingulate gyrus; posterior division (28.1%); precuneous cortex (47.3%)
580	5.22	−48	−64	50	Angular gyrus (18.3%); lateral occipital cortex, superior division (38.5%)
465	4.94	−40	10	60	Superior frontal gyrus (15.7%); middle frontal gyrus (25.6%)
189	4.15	16	−86	−40	Right Crus I (48.0%); right Crus II (44.2%)
179	4.44	−68	−40	−4	Superior temporal gyrus, posterior division (17.2%); middle temporal gyrus; posterior division (40.5%); middle temporal gyrus, temporo-occipital part (14.9%)
158	4.45	−44	48	−6	Frontal pole (73.2%)
105	5.07	−16	60	16	Frontal pole (59.1%)
74	3.98	52	−64	34	Angular gyrus (14.7%); lateral occipital cortex; superior division (45.5%)
70	3.94	−2	54	−8	Frontal pole (12.3%); frontal medial cortex (41.2%); paracingulate gyrus (30.6%)
68	4.22	−30	−14	70	Precentral gyrus (35.8%)
54	4.73	−38	−74	−36	Left Crus I (84.5%); left Crus II (11.2%)
52	4.16	44	−66	44	Lateral occipital cortex, superior division (64.4%)
48	4.26	8	−82	−26	Right Crus I (66.8%); right Crus II (27.1%)
35	3.93	−52	28	16	Middle frontal gyrus (12.9%); inferior frontal gyrus, pars triangularis (45.4%)

*^a^*The maximum *Z* score within each cluster and its location in relation to the MNI brain atlas are shown. The anatomical location of the cluster determined with Autoaq (part of FSL software), based on the degree of overlap with probabilistic atlases (Harvard Oxford Cortical Structural Atlas, Harvard Oxford Subcortical Structural Atlas, Cerebellar Atlas in MNI152 space after normalization with FNIRT), is given. Only those structures to which the cluster had a >10% chance of belonging to are presented.

### Attention-mediated analgesia (brainstem-focused analysis)

**Figure 6.**
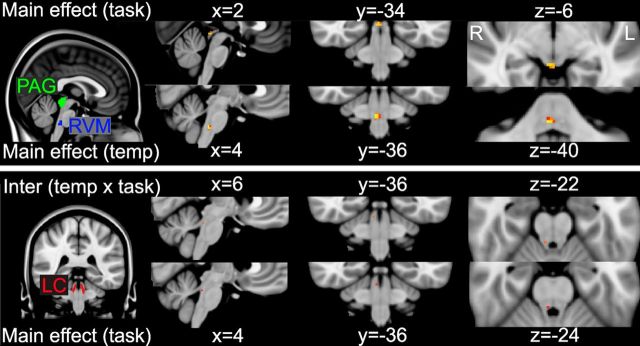
Group main effects of temperature, task, and their interaction in the PAG, LC, and RVM, as assessed by nonparametric permutation testing within anatomically defined masks (described in Fig. 2). Top row, Location of the PAG and RVM masks, and the activity observed within the main effect of task (dorsal PAG) and temperature (RVM). Bottom row, Location of the LC running parallel to the edges of the fourth ventricle, and the activity within the right LC during the main effect of task, and a task × temperature interaction. Slice locations are given for each condition in MNI coordinates for the voxel with lowest *p* value surviving correction for multiple comparisons, based on TFCE (*p* < 0.05). Voxel color represents significance level: red represents 0.05; yellow represents 0.001.

At the whole-brain corrected level, no activity was seen within the brainstem for any contrast. Therefore, given the known signal-to-noise issues associated with brainstem imaging, to assess responses within the brainstem structures, a focused analysis using anatomical masks was performed. The masks obtained by gray matter probability segmentation of the T2-weighted volume scan can be seen in Figure 2, highlighting the location of the PAG, LC, and RVM (and also in Fig. 6). Within these objectively defined probabilistic masks, main effects and their interaction were assessed with permutation testing, and are reported as TFCE corrected *p* < 0.05. This analysis showed a main effect of task (hard > easy) within the dorsal PAG and a main effect of temperature (high > low) in the RVM (Fig. 6, top row). In addition, a main effect of task and an interaction between task and temperature were observed in the LC, contralateral to the thermal stimulation (Fig. 6, bottom row).

### Intrasubject parametric regression with pain ratings

**Figure 7.**
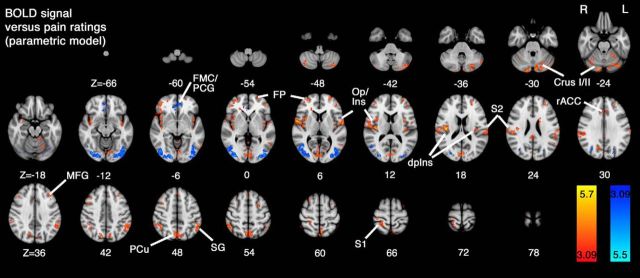
Whole-brain analysis of intrasubject parametric regression obtained from pain ratings and BOLD signal measured across the 5 experimental conditions. Results from a mixed-effects one-sample group average model demonstrate regions where activity scales linearly in a positive direction (red-yellow) and in a negative direction (blue-light blue). Notably, the peak *Z* score was observed to lie in the dpIns, but also extended into the adjacent parietal operculum (Op)/S2 region represented bilaterally. Other regions demonstrating a linear relationship with pain ratings include areas of prefrontal cortex, cerebellum, supramarginal gyrus (SG), S1, and precuneus (PCu). Areas whose activity decreased in line with pain ratings included visual association areas (lateral occipital cortices [LOC]) and FMC/PCG. Data were obtained from cluster-based thresholding using an initial threshold of *Z* > 3.09 and corrected significance level of *p* < 0.05.

Brain regions whose activity was linearly related to perceived pain intensity were identified using an intrasubject parametric regression model. Brain areas showing a similar relationship across the group (determined using a mixed-effects model and one-sample *t* test) were grouped according to whether the BOLD versus pain rating relationship was positive or negative (Fig. 7). The area showing strongest positive relationship between perceived pain intensity and BOLD signal amplitude was the dorsal posterior insula contralateral to the side of stimulation. Similar effects were also observed in the contralateral primary somatosensory cortex, and bilaterally in S2/operculoinsular cortices (i.e., similar to the areas identified in the positive main effect of temperature; Fig. 4). A positive relationship to pain ratings was also observed within the precuneus, bilateral parietal association cortices, and cerebellum. A negative relationship between pain ratings and BOLD signal was observed in visual association cortices, which was similar to the pattern of activity observed in the positive main effect of task (Fig. 5), and in ventromedial prefrontal cortex, similar to the negative main effect of temperature (Fig. 4). Coordinates, *Z* scores, and anatomical location for the clusters identified from the parametric regression are summarized in Table 2.

**Table 2.** Data from parametric regression analysis obtained with cluster-forming threshold *Z* > 3.09 and (corrected) *p* < 0.05*^a^*

Voxels	Maximum *Z*	*x* (mm)	*y* (mm)	*z* (mm)	Atlas label(s)
Positive slope for relationship between BOLD signal amplitude and pain ratings					
1756	5.7	38	−16	20	Insular cortex (12.0%); central opercular cortex (14.6%)
1439	4.45	−10	−82	−30	Lingual gyrus (10.9%); left Crus I (25.9%); left Crus II (19.3%)
491	4.33	−4	−74	48	Precuneous cortex (54.1%)
423	5.05	−60	−2	6	Insular cortex (25.5%); central opercular cortex (17.6%)
385	4.35	−50	−46	46	Supramarginal gyrus, posterior division (34.2%); angular gyrus (18.6%)
236	4.18	28	58	6	Frontal pole (64.4%)
234	4.83	26	−40	70	Postcentral gyrus (32.4%); superior parietal lobule (22.3%)
180	4.71	−66	−20	28	Postcentral gyrus (14.8%); supramarginal gyrus, anterior division (18.4%); parietal operculum cortex (22.0%)
173	4.51	14	−84	−24	Right Crus I (36.2%); right Crus II (46.3%)
173	4.22	−24	56	−2	Frontal pole (72.4%)
125	4.35	16	−66	34	Precuneous cortex (38.2%); cuneal cortex (17.0%)
89	4.1	−20	−44	−24	Cerebellum: Left I-IV (20.6%); left V (40.5%); left VI (32.5%)
83	4.07	0	28	44	Superior frontal gyrus (39.2%)
79	4.06	2	−38	22	Cingulate gyrus, posterior division (65.2%)
79	4.24	36	−58	−28	Cerebellum: Right VI (49.0%); right Crus I (49.7%)
76	4.12	−62	−60	−6	Middle temporal gyrus, temporo-occipital part (46.1%); lateral occipital cortex, inferior division (16.4%)
73	4.17	−32	32	42	Middle frontal gyrus (63.3%)
66	4.07	−16	2	24	Left lateral ventricle (15.5%); left caudate (31.6%)
59	4.2	−30	46	24	Frontal pole (76.8%)
56	4.11	2	26	28	Paracingulate gyrus (26.7%); cingulate gyrus, anterior division (57.1%)
54	4.35	−42	44	0	Frontal pole (69.0%)
53	4.07	46	10	42	Middle frontal gyrus (38.0%)
49	3.97	36	−72	−48	Right Crus II (75.1%)
46	4.09	40	28	40	Middle frontal gyrus (59.9%)
44	4.1	20	30	4	Right cerebral white matter
44	4.26	2	−50	−4	Cerebellum: Left I-IV (22.2%); right I-IV (24.9%)
38	3.85	−32	2	66	Superior frontal gyrus (11.1%); middle frontal gyrus (32.8%)
38	4.21	−22	8	54	Superior frontal gyrus (26.4%); middle frontal gyrus (19.4%)
34	4.39	−36	4	12	Insular cortex (23.7%); central opercular cortex (35.1%)
31	3.91	34	−80	−24	Right Crus I (93.1%)
30	4.05	26	20	60	Superior frontal gyrus (33.7%); middle frontal gyrus (14.3%)
29	3.94	−54	−58	22	Angular gyrus (36.5%); lateral occipital cortex, superior division (33.3%)
Negative slope for relationship between BOLD signal amplitude and pain ratings					
1102	5.5	20	−88	−10	Lateral occipital cortex, inferior division (35.5%)
957	4.55	−42	−88	−8	Lateral occipital cortex, inferior division (42.3%)
190	4.51	−8	44	−6	Frontal medial cortex (18.9%); paracingulate gyrus (29.6%); cingulate gyrus, anterior division (11.9%)
53	4.28	32	−66	30	Lateral occipital cortex, superior division (48.5%)
34	3.91	−26	−74	28	Lateral occipital cortex, superior division (50.6%)

*^a^*The maximum *Z* score within the cluster and its location in relation to the MNI standard brain atlas are shown. The anatomical location of the cluster determined with Autoaq (part of FSL software), based on the degree of overlap with probabilistic atlases (Harvard Oxford Cortical Structural Atlas, Harvard Oxford Subcortical Structural Atlas, Cerebellar Atlas in MNI152 space after normalization with FNIRT), is given. Only those structures to which the cluster had a >10% chance of belonging to are presented.

**Figure 8.**
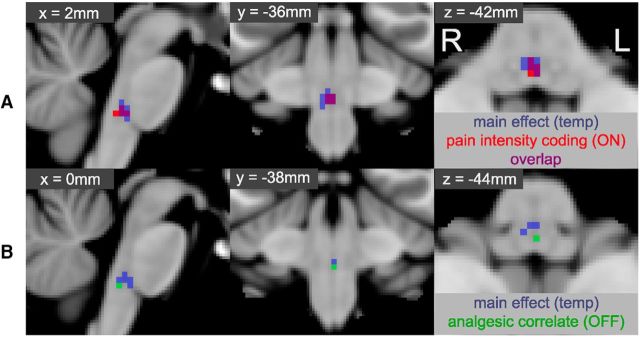
Intrasubject and intersubject parametric regression of pain ratings and analgesia. Top row (***A***) represents the mixed-effects one-sample group average for the intrasubject parametric regression model using pain ratings and BOLD signal within the RVM (red). For comparison, the main effect of temperature for the RVM is show in blue, and the overlap depicted in purple. There is clear overlap between the area of activity identified in the main effect of temperature, and those voxels whose activity scaled linearly (up) with increasing pain ratings, suggestive of a role in pain intensity coding as has been observed with ON-cell activity. Bottom row (***B***) represents the equivalent intersubject parametric regression obtained using the difference in pain scores (ΔVAS = easy − hard, during high temperature stimulation) and the difference in BOLD signal (hard − easy, during high temperature stimulation). The region identified as reflecting magnitude of analgesic effect (green) lies within the RVM mask but is caudal to that responding linearly to increasing pain ratings. Activity that scales with the reported magnitude of pain reduction is suggestive of an analgesic role, which has previously been associated with OFF-cell activity. All data obtained by permutation testing with an RVM mask, and “activated” voxels reported for TFCE-corrected *p* < 0.05. Voxel coordinates are in MNI space and reflect the locations of the voxel with lowest *p* value for parametric pain intensity coding (***A***) and analgesic correlation (***B***).

Within the brainstem, we observed a positive relationship between pain ratings and BOLD signal only in the RVM (see Fig. 8*A*), in a region that almost completely coincided with that identified in the positive main effect of temperature (see overlap on Fig. 8*A*).

### Intersubject regression with analgesia ratings

A whole-brain mixed-effects comparison between the hard|high and easy|high conditions revealed that, at the chosen threshold, the magnitude of the paired difference in BOLD signal was not significantly linearly related to the average intersubject differences in pain ratings (i.e., analgesia scores) for any region. To explore whether the magnitude of pain relief due to attention was related to activity with the brainstem, a masked analysis was performed using permutation testing. A positive relationship between ΔVAS and BOLD signal difference was observed within the RVM (i.e., decreased pain ratings were associated with increased BOLD activity). This area was caudal and distinct from the RVM clusters identified in the main effect of pain/intrasubject parametric regression (see Fig. 8*B*).

## Discussion

It has long been appreciated that different aspects of the pain construct are represented in distinct forebrain regions. In this study, we have demonstrated that attentional analgesia differentially involves specific brainstem structures. We observe distinct activity profiles within regions of the RVM: one area where activity increases in response to high temperature stimulation and is linearly related to pain intensity, and another region where activity increases proportionally with analgesic effect. We replicate previous findings demonstrating involvement of the PAG in attention processes and show that it is activated during times of high attentional load (Tracey et al., 2002; Valet et al., 2004). Additionally, we find that the contralateral LC is active during the main effect task and is the only area whose activity shows a task × temperature interaction. Finally, using intrasubject regression, we show that activity within forebrain areas, including the dorsal posterior insula, encodes perceived pain intensity. Our findings support the principle that attentional analgesia recruits a descending pain control PAG-RVM/LC system in the brainstem.

The PAG is known to be a key integrator and orchestrator of behavioral selection and an interface between higher cognitive centers and brainstem/spinal motor, sensory, and autonomic control territories. Within our paradigm, activity in the dorsomedial region of the PAG was seen as a main effect of task. A similar activation of the PAG by attention/distraction has been reported in studies of attentional analgesia (Tracey et al., 2002; Valet et al., 2004). The PAG, anterior insular, and frontal cortices are commonly activated during cognitive tasks, similar to the network engaged here, and are thought to represent salience and executive control (Seeley et al., 2007). Unlike Tracey et al. (2002), we did not find any relation between PAG activity and analgesic action. However, as discussed below we did find such a relationship for an RVM cluster so are confident that we had the power to detect such an effect in the PAG. We believe that our finding may reflect the role of the PAG as an integrator (Roy et al., 2014) of programmed behavior (here potentially promoting task performance by attenuating nociceptive transmission) and that attenuation of nociception is enacted by downstream agents, such as the RVM and the LC (Fields, 2000; Ossipov et al., 2010).

The RVM has been extensively investigated in animal models and shown to be a key site for integration of ascending nociceptive information and a source of descending control (Fields, 2000; Ossipov et al., 2010). Within this region are pronociceptive neurones (“ON-cells”) that are activated by noxious stimuli and a population of antinociceptive neurones (“OFF-cells”) that are inhibited by noxious stimuli. In our study, we have seen patterns of activity consistent with the predicted behavior of these two pools of neurones: one pronociceptive cluster activated by hot stimuli where activity scaled linearly with pain perception; and a second more caudally located cluster whose activity is linked to an antinociceptive effect of attention. Two previous pain fMRI studies have reported activity within a similar brainstem region to our RVM mask (Fairhurst et al., 2007; Eippert et al., 2009). To our knowledge, the current study is the first to uncover supportive evidence for the existence of distinct clusters of pronociceptive and antinociceptive neurones in the human RVM. Further, this provides a missing link between the activation of the PAG and spinal modulation of nociceptive processing (Sprenger et al., 2012).

Within the contralateral LC, we found activation in response to both the main effect of task and a task by temperature interaction. Notably, this was the only region in the brainstem (or whole brain) that mirrored the interaction between pain and attention seen in behavioral pain ratings. Cellular LC recordings in rodents have shown that these neurones respond to noxious stimulation of the contralateral paw with a brief burst of activity (Cedarbaum and Aghajanian, 1978; Sugiyama et al., 2012). This lends credence to our fMRI finding of a lateralized interaction between task and temperature as being consistent with the known functional organization of the LC. This finding may also be consistent with the LC playing a role in the analgesic effect, as our study also found that activity within the contralateral LC may reflect the magnitude of analgesia, although the response did not reach significance (*p* = 0.07).

We observed main effects of task difficulty in a wide range of cortical structures known to be involved in visual perception (Ganis et al., 2004) and broader attention processing (Petersen and Posner, 2012). Task-negative responses were observed in regions belonging to the default mode network (Damoiseaux et al., 2006). The main effect of temperature, when subjects perceived stimulation as painful, was seen in brain regions previously reported to be associated with pain (Apkarian et al., 2005; Tracey and Mantyh, 2007). Our design permitted regression of intrasubject pain ratings and individual BOLD responses via parametric analysis (Büchel et al., 1998). This revealed that the most prominent brain region involved in pain perception/intensity encoding was the dorsal posterior insula. These results build on earlier findings demonstrating that dpIns is involved in the sensory-discriminative aspects of pain (Brooks et al., 2002, 2005), whose activity tracks pain perception (Segerdahl et al., 2015a) and where electrical stimulation can produce pain (Mazzola et al., 2009). It should be noted that others have questioned whether dpIns activity can be attributed specifically to nociception (Mouraux et al., 2011; Davis et al., 2015; Segerdahl et al., 2015b; Liberati et al., 2016). Our contribution to this ongoing debate is to note that, when controlling for nonspecific effects of thermal stimulation and using a salient attention task within a 2 × 2 factorial design, we find dpIns activity that (1) can plausibly be attributed to pain perception and (2) scales linearly with pain ratings.

We observed activity in the FMC and PCG in the negative main effect of temperature (i.e., warm > hot), that overlaps with the area negatively correlated with perceived pain. This region has previously been reported as “subgenual rACC” (e.g., Eippert et al., 2009; Palomero-Gallagher et al., 2015) and has been shown to deactivate in response to high temperature (painful) stimulation (Schoell et al., 2010), as also shown in this study. The authors related this finding to the presumed inhibitory effect of endogenous opioids, which they could reverse with naloxone. Furthermore, connections between the subgenual rACC and PAG have been demonstrated in humans using probabilistic tractography (Wang et al., 2014).

There have been several recent debates in the literature about the reliability of MRI localization of brainstem structures, such as the PAG and LC (Astafiev et al., 2010; Linnman et al., 2012). These issues arise because of the technical challenge of imaging the brainstem (Brooks et al., 2013) and the lack of a validated probabilistic atlas. Although work is underway to develop multimodal segmentation approaches for the human brainstem (Lambert et al., 2013), there is a pressing need for a probabilistic atlas defined on the basis of imaging (Faull et al., 2015) and histology/immunocytochemistry (Eickhoff et al., 2010). We have attempted to address this issue by segmenting brainstem gray matter from T2-weighted structural scans and using this information to generate PAG, LC, and RVM masks. The localization of our PAG mask is within the area of maximum PAG activation identified by Linnman (2012); see also recent functional PAG segmentation (Ezra et al., 2015). Similarly, our LC mask sits in the same region identified using neuromelanin contrast on MRI (Keren et al., 2009). Our RVM region overlaps with the tissue class identified as “monoaminergic” (Lambert et al., 2013) and is anatomically consistent with the nucleus raphe magnus (Naidich et al., 2009).

Concerning the specificity of findings, we undertook additional exploratory analyses using a whole brainstem mask (Harvard-Oxford subcortical atlas, 60% threshold). This provided evidence supporting a main effect of temperature in the RVM (*p* < 0.05 corrected). The only other areas activated in this condition were the dorsolateral pontine tegmentum and nucleus of the solitary tract, structures known to be involved in pain processing, but outside our originally hypothesized regions of interest. All activity was contralateral to the side of stimulation. No other contrast gave rise to activity that survived threshold at *p* < 0.05 corrected using the whole brainstem mask.

In conclusion, we used a brainstem optimized whole-brain imaging protocol, adjusted for geometric distortions via field maps and corrected data for the influence of physiological noise (Brooks et al., 2013). To control for nonspecific effects of stimulus application and arousal, we used a 2 × 2 factorial design. We were able to demonstrate cortical areas, such as the dpIns, whose activity appears to be linearly related to the amount of pain perceived. The modulation of pain perception by attentional load is reflected in the brainstem in the PAG (attention), LC (attention and interaction between task and temperature), and most strikingly at the level of the RVM, which demonstrates both a pain-encoding profile, potentially reflecting activity of ON-cells, and an anatomically distinct graded response that is correlated to the magnitude of analgesia (consistent with OFF-cell activity). This indicates the engagement of a descending midbrain-pontine-medullary circuit in this attentional analgesia paradigm. Future work will explore the nature of brainstem–cerebrum connectivity and its relationship to the magnitude of attentional analgesia.

It remains to be demonstrated whether the RVM and LC are indeed directly regulating spinal nociceptive transmission in human, which is likely to require dedicated hardware (e.g., custom brainstem/spinal cord coils) and sequence optimization (Finsterbusch et al., 2013; Sprenger et al., 2015). This paradigm and imaging approach may provide a means to objectively test whether the long hypothesized aberrant balance in the function of these important brainstem endogenous analgesic circuits is seen in chronic pain conditions as suggested by (Edwards, 2005) and whether this can indeed account for the increased risk of developing chronic pain (Yarnitsky et al., 2008).
